# Health-Related Quality of Life Among Type 2 Diabetes Patients With Depressive Symptoms in Vietnam

**DOI:** 10.1155/jdr/6992121

**Published:** 2025-03-28

**Authors:** Nguyen Tran Kien, Nguyen Phuong Hoa, Ha Huu Tung, Kris Van den Broeck, Johan Wens

**Affiliations:** ^1^Family Medicine Department, Hanoi Medical University, Hanoi, Vietnam; ^2^Faculty of Medicine and Health Sciences, University of Antwerp, Antwerp, Belgium; ^3^General Hospital of Agriculture, Hanoi, Vietnam

**Keywords:** depression, diabetes, quality of life, Type 2 diabetes mellitus, Vietnam

## Abstract

**Background:** This study investigates the impact of Type 2 diabetes mellitus (T2DM) and depressive symptoms on the health-related quality of life (HRQoL) among patients at the Agricultural General Hospital in Hanoi, Vietnam. The research explores the interconnections between chronic physical conditions and mental health within a resource-constrained healthcare environment.

**Methods:** A cross-sectional survey was conducted with 516 T2DM patients using the SF-36 to assess HRQoL and the PHQ-9 to measure depressive symptoms. The study examined the prevalence of depressive symptoms and their correlation with various HRQoL components.

**Results:** Among the participants, 45.2% exhibited depressive symptoms from mild to severe levels. Significant disparities in HRQoL scores were observed, particularly in physical composite and overall quality of life scores between T2DM with and without depressive symptoms. Statistical analysis highlighted that depressive symptoms significantly diminish HRQoL, with the PHQ-9 scores serving as a robust predictor.

**Conclusion:** The findings underscore the critical need for integrated care approaches that include mental health support for T2DM patients. Routine screening for depressive symptoms should be a component of diabetes management protocols to improve overall patient outcomes. Further longitudinal research is needed to confirm these findings and develop effective interventions.

## 1. Introduction

Diabetes mellitus stands as a pervasive chronic noncommunicable disease, undergoing a rapid escalation from 108 million individuals in 1980 to approximately 537 million worldwide in 2021 [1, 2]. Particularly alarming is the prevalence among the elderly, with one in five individuals aged 65 and above grappling with diabetes, primarily concentrated in low- and middle-income countries [3, 4]. Acknowledged by the World Health Organization [5] as the most prevalent form, Type 2 diabetes mellitus (T2DM) not only inflicts severe health-related complications but also precipitates a substantial diminution in the affected individuals' quality of life [6, 7].

Health-related quality of life (HRQoL), a multifaceted construct encompassing an individual's perceptions of physical, emotional, and social well-being [8, 9], is notably compromised in individuals contending with T2DM due to the exigencies of treatment [10, 11]. Given the chronic nature of diabetes, achieving a complete cure is an unattainable objective, underscoring the importance of maintaining patients at a stable stage, both physically and mentally. The identification of predictors and risk factors for HRQoL assumes paramount significance, as it informs the development of comprehensive interventions and treatments for those living with diabetes [12].

The assessment of HRQoL among adults with diabetes has emerged as a pressing global topic, spanning research endeavors in the United States [13], Europe [14, 15], and Asia [16–18]. Numerous factors, including sex, age, income, comorbidities, and lifestyle, have been correlated with HRQoL in adults with diabetes [16–18].

Additionally, the intricate relationship between T2DM and depression has garnered substantial attention, revealing a bidirectional association. Research, such as that conducted by Nguyen et al. and Mezuk et al., underscores the heightened susceptibility of individuals with diabetes to depression and vice versa [19, 20]. Chronic stress stemming from diabetes management has been identified as a contributory factor to depression, with studies by Fisher et al. illuminating the heightened stress levels associated with the complexities of diabetes self-care [21].

In the context of Vietnam, a developing nation witnessing a precipitous rise in diabetes incidence [22], the impending prevalence of diabetes and prediabetes, projected to reach 7% and 15.7% of the total population by 2035, respectively, underscores the urgency of addressing this health concern [23]. Despite this, empirical evidence concerning HRQoL among adults living with T2DM, particularly those with depressive symptoms, remains scarce in resource-constrained settings like Vietnam [24, 25]. Consequently, this study examined the specific impact of depressive symptoms on different domains of HRQoL among T2DM patients in Hanoi, Vietnam. Depressive symptoms are hypothesized to significantly lower HRQoL scores across physical and mental health (MH) domains. Identifying these impacts provides valuable insights into the importance of integrating mental healthcare within diabetes management protocols. Additionally, the study addresses the scarcity of empirical evidence on HRQoL among T2DM patients with depressive symptoms in resource-constrained settings like Vietnam, highlighting the bidirectional relationship between diabetes and depression.

## 2. Methodology

### 2.1. Study Setting

Established in 2010 with the support of Liege University, Belgium [26], the Family Medicine Centre (FMC) initially operated as the first family doctor center in Hanoi. Subsequently, the FMC was amalgamated into the outpatient department, where patients with diabetes are currently managed [27]. The selection of the FMC was predicated on its accessibility, the presence of trained personnel, and a substantial database of individuals with diabetes.

### 2.2. Study Design

In 2020, a cross-sectional interviewer-administered survey was conducted among out-clinic patients at the FMC of the Agricultural General Hospital in Hanoi, Vietnam, to inventory participants' state and level of depression and HRQoL. We hypothesized that depressed patients with T2DM would experience lower HRQoL compared to patients with T2DM and without depression. Further analyses were explorative in nature.

The data collection team comprised four well-trained undergraduate medical students, a deliberate choice to mitigate social desirability bias during the data collection process. Notably, medical staff within the hospital setting were deliberately excluded from the data collection team. Patient interviews were conducted immediately following the diabetic examination and administration of prescribed medications. This methodological approach aimed to ensure an unbiased and candid acquisition of information from the study participants.

### 2.3. Sampling

The sample size was determined based on prevalence data for depressive symptoms among diabetic populations. Drawing on the insights of Hermanns et al., who reported that approximately 14.1% of diabetic patients exhibit depressive symptoms, we determined that a sample size of 517 patients was necessary with a significance level (*α*) of 0.05 and a margin of error (*ε*) set at 0.03 [28]. Anticipating potential refusals to participate, we extended invitations to 519 patients diagnosed with Type 2 diabetes. The selection of participants was randomized from the pool of patients slated for routine follow-up appointments.

### 2.4. Study Participants

The inclusion criteria for participants in this study comprised individuals who (i) were aged 18 years or older; (ii) had received a diagnosis of Type 2 diabetes at least 6 months before the commencement of the study, in accordance with the “Guidelines for Diagnosis and Treatment of Type 2 Diabetes” issued by the Vietnamese Ministry of Health in 2017 (Decision No. 3319/QD-BYT); (iii) were registered as outpatients undergoing diabetes treatment in the Department of Outpatient at Agricultural General Hospital; and (iv) possessed the ability to read, write, and communicate effectively with the interviewer [29]. Patients with severe medical conditions or those unwilling to participate were excluded from the study. Prior to involvement, eligible patients were requested to provide written informed consent.

### 2.5. Measurements and Instruments

Preceding the primary survey, a preliminary pilot study was conducted with a cohort of 20 participants characterized by diverse socioeconomic attributes, aimed at assessing the content validity of the questionnaire. Minor adjustments to the wording were implemented in response to the feedback received from the participants. The questionnaire encompassed the following informational domains.

*Socioeconomic characteristics*. Participants self-disclosed information related to demographic variables, including gender (male/female), age, occupational status (employed, unemployed, and retired), educational attainment (primary school or lower, secondary school, and high school or higher), and body mass index (BMI) categorized as < 23, ≥ 23–< 25, and ≥ 25.

*Diabetes treatment–related characteristics*. Participants were queried regarding their health risk behaviours, encompassing inquiries into smoking habits, alcohol consumption, and levels of physical activity. Additionally, the questionnaire was structured to investigate essential aspects such as the duration of diabetes, presence of comorbidities, proximity to the nearest health facility, health insurance coverage, and self-care proficiency.

*Psychological measurement using Short Form 36 (SF-36) questionnaire*. The widely employed SF-36 health survey questionnaire serves as a prevalent instrument for assessing quality of life. In Vietnam, the SF-36 questionnaire has been extensively utilized after undergoing thorough validation for use in Vietnam. This included forward and backward translation, pilot testing with representative populations, and statistical validation processes [30]. Comprising eight dimensions, the SF-36 assesses various facets of well-being, including physical functioning (PF), role limitations due to health problems (RP), bodily pain (BP), general health perceptions (GH), vitality (VT), social functioning (SF), role limitations due to personal or emotional health problems (RE), and general MH. The initial four dimensions are aggregated to derive the physical composite score (PCS), while the latter four dimensions contribute to the calculation of the mental composite score (MCS). SF-36 total scores range from 0 to 100, with higher scores indicative of a superior quality of life.

#### 2.5.1. PHQ-9 (Patient Health Questionnaire-9)

The PHQ-9, a screening tool for depression severity, was similarly validated for use in Vietnam through rigorous processes. The validation steps involved cultural adaptation, pilot testing, and psychometric analyses to confirm its reliability and construct validity. The Vietnamese version of the PHQ-9 has demonstrated strong internal consistency and diagnostic accuracy in identifying depressive symptoms in various populations, including individuals with chronic illnesses. Its use in this study ensures alignment with established methodologies for assessing MH in Vietnam. Comprising nine items, each scored from 0 to 3, the total PHQ-9 score spans from 0 to 27. Depression severity was classified using predefined cut-offs: *normal* (0–4 points), *mild* (5–9 points), *moderate* (10–14 points), *moderate-severe* (15–19 points), and *severe* (≥ 20 points). Patients with a score exceeding 4 were categorized as “having depressive symptoms.”

### 2.6. Statistical Analysis

Statistical analysis was performed using Statistical Package for the Social Sciences 23.0 (SPSS Inc., Chicago, IL, United States). Descriptive statistics were used to summarize the patients' characteristics. An independent samples *t*-test or Mann–Whitney *U* test was used for group comparisons. Pearson's correlation was performed to test the correlation among continuous variables, while Spearman's correlation was performed among ranked variables. Because the outcome data were normally distributed, multivariate linear regressions were applied to examine factors associated with psychological problems. All statistical tests were two-sided (*α* = 0.05). *p* value < 0.05 was accepted as significant.

### 2.7. Ethics Statement

The study received ethical approval from the scientific council of the General Hospital of Agriculture, which granted approval on October 1, 2019. All participants were assured of confidentiality, asked to sign an informed consent, and given instructions informing them about the ability to refuse to answer any question or discontinue their participation at any time. Patients' personal information collected from interviews was encoded, and only researchers had the ability to access the data. All data was used for research purposes only.

## 3. Result

Table 1 outlines the characteristics of depressive symptoms among patients with T2DM. Approximately 36.0% of participants exhibited mild depressive symptoms, while around 10% scored at or above 10 on the PHQ-9, indicating a more severe condition. The prevalence of moderate to severe depressive symptoms was 7.6%, 1.4%, and 0.2%, respectively. The mean PHQ-9 score was 5.0 (SD = 2.9), placing it within the “mild” symptom range.

Table 2 presents a comparative analysis of sociodemographic, clinical, and behavioural characteristics among 516 T2DM patients with and without depressive symptoms. The table includes data on gender distribution, age, education levels, and occupational status under sociodemographic factors. Clinical characteristics encompass BMI, comorbidities, disease duration, and medication adherence. Behavioural factors, including smoking and alcohol consumption, are also documented.

The mean age of participants was 67 years (SD = 9.0), with those experiencing depressive symptoms being slightly younger than their counterparts without depressive symptoms (66 vs. 68 years, *p* = 0.03 < 0.05). Educational attainment was relatively similar across groups, with nearly half of the participants having completed secondary school. Occupational status exhibited no statistically significant variation between groups (*p* = 0.30), with 30.3% of patients with depressive symptoms and 32.6% of those without depressive symptoms being employed, while 62.8% and 63.5%, respectively, were retired.

Regarding clinical characteristics, BMI was comparable between groups (22.8 vs. 22.9, *p* = 0.70). Insulin injection use was also examined, with 18.8% of participants reporting insulin use. The proportion of insulin users was slightly higher among those with depressive symptoms (20.8%) compared to those without (17.2%), though this difference was not statistically significant (*p* = 0.34). However, a slightly higher proportion of patients without depressive symptoms reported comorbidities (93.1% vs. 88.1%, *p* = 0.09). Disease duration did not significantly differ between groups (*p* = 0.13), with patients experiencing depressive symptoms having a mean duration of 8.6 years (SD = 6.9) compared to 7.6 years (SD = 6.1) for those without depressive symptoms. Notably, adherence to treatment, measured by medication adherence in the past month, showed a slight difference, though not statistically significant (*p* = 0.28).

Behavioural characteristics revealed that smoking prevalence was not significantly different between groups (*p* = 0.27), though alcohol consumption showed a statistically significant variation (*p* = 0.04), with a higher proportion of patients without depressive symptoms reporting current alcohol use.

Table 3 highlights the scores of the eight domains of HRQoL of study participants between patients with and without depressive symptoms. There were significant statistical differences between the scores of T2DM patients with and without depressive symptoms in five out of the eight SF-36 domains, including PF, role limitations due to physical health, VT, MH, and BP. Besides, PCS and overall quality of life scores were statistically significantly different as well. In general, in T2DM patients without depressive symptoms, overall HRQoL was statistically higher than in T2DM patients with depressive symptoms (63.03 ± 14.20 vs. 60.31 ± 14.30, *p* = 0.031 < 0.05). The PCS of T2DM patients with depressive symptoms was lower compared to patients without depressive symptoms (57.68 ± 19.68 compared to 61.98 ± 19.68, *p* = 0.014 < 0.05), indicating a lower physical HRQoL. Meanwhile, no significant difference was observed between the MCSs of T2DM patients with and without depressive symptoms (62.95 ± 12.91 vs. 64.08 ± 11.66, *p* = 0.294). This suggests that while physical aspects of HRQoL are statistically impacted by depression, the overall mental component requires further exploration. Given that SF-36's MH domain reflects broader psychological factors, we further examined its correlation with PHQ-9 scores.

In testing for correlation between HRQoL scores and other factors (Table 4), a significant correlation has been found between both PCS and MCS as well as overall score and PHQ-9, with *p* < 0.001. In addition, there was a statistically significant relationship between HRQoL scores and age.

Multivariate regression analysis revealed significant associations between HRQoL and various predictors (Table 5). Higher PHQ-9 scores were strongly associated with lower PCS (*β* = −1.078, 95% CI: −1.762; −0.395, *p* < 0.01) and overall HRQoL (*β* = −0.685, 95% CI: −1.181; −0.188, *p* < 0.01), indicating that an increase in depressive symptom severity significantly diminishes physical and overall quality of life. Age was another key predictor, with older participants experiencing lower PCS and MCS scores (*β* = −0.734 and −0.371, respectively, both *p* < 0.001). These findings suggest that depressive symptoms exacerbate the natural decline in HRQoL associated with aging, necessitating targeted interventions for older adults with diabetes.

Conversely, being employed and having a higher level of education were associated with improved PCS and overall HRQoL scores. For example, participants with secondary or higher education scored, on average, 6 points higher on PCS (*β* = 5.951, 95% CI: 0.631; 11.270, *p* < 0.05) and nearly 4 points higher on overall HRQoL (*β* = 3.965, 95% CI: 0.104; 7.826, *p* < 0.05) compared to those with lower education levels. These results suggest that socioeconomic advantages play a protective role, possibly through better access to healthcare resources and enhanced self-management capabilities.

Meanwhile, there are no significant relationships between BMI, diabetes duration, comorbidity, and insulin injection with physical, mental, and overall quality of life scores.

## 4. Discussion

This study highlights the significant interplay between depressive symptoms and HRQoL among patients with T2DM in Hanoi, Vietnam. Our findings illuminate how depressive symptoms severely impact both the physical and emotional well-being of diabetic patients, aligning with existing literature that notes a substantial decline in HRQoL among patients with concurrent depressive symptoms [9, 31]. Significantly, depressive symptoms were found to be a robust predictor of reduced HRQoL across several domains, including PF, emotional well-being, and overall life satisfaction [16, 17].

Age also emerged as a significant factor affecting HRQoL in our study. Older patients and those with multiple health conditions demonstrated lower HRQoL scores, suggesting that the burden of managing T2DM intensifies with age and additional health complexities [19, 20, 32]. This finding underscores the need for targeted interventions that address the compounded challenges faced by elderly T2DM patients, particularly in Vietnam, where the aging population is growing. The bidirectional relationship between depression and diabetes further complicates disease management, reinforcing the necessity of routine screening for depressive symptoms and tailored psychological interventions.

Furthermore, the bidirectional relationship between depression and diabetes is particularly pronounced, as evidenced by significant correlations with both PCS and MCS. This complex interplay appears to be driven by a combination of physiological and behavioural mechanisms [4]. Physiologically, chronic inflammation, frequently elevated in individuals with T2DM, has been implicated in both depressive symptoms and physical health declines. Inflammation can disrupt various metabolic and neural pathways, exacerbating diabetes complications and impairing HRQoL. Additionally, alterations in hypothalamic–pituitary–adrenal (HPA) axis activity, commonly associated with depression, may amplify the stress response, further compounding the burden of diabetes and its management [33, 34].

Behaviorally, depressive symptoms can reduce motivation and adherence to essential self-care practices such as medication compliance, regular physical activity, and glucose monitoring [35, 36]. These lapses in self-management not only worsen diabetes control but also contribute to declines in PF and VT, as observed in this study. Lower PCSs among individuals with higher depressive symptom severity underscore the tangible impact of psychological distress on physical health outcomes [37].

Besides, this relationship is likely driven by the chronic stress associated with diabetes management, which exacerbates depressive symptoms, thereby forming a detrimental cycle that impacts overall diabetes care and patient well-being [38]. This cyclic effect is exacerbated by the stress and challenges of daily diabetes management, suggesting a compounded impact on older adults who may already be dealing with age-related health declines. This interplay points to the critical need for integrated care approaches that simultaneously address diabetes management and MH support, specially designed to break this cycle and improve overall outcomes for all age groups, with particular attention to the elderly [7, 39]. Such interventions could significantly improve quality of life by reducing the dual burden of disease and depressive symptoms, offering a more supportive and effective management strategy for aging populations living with T2DM.

Interestingly, our study found that higher levels of education and employment status correlated positively with improved HRQoL scores among patients with T2DM. This suggests that socioeconomic advantages generally contribute to better overall health outcomes and might provide better access to healthcare resources, healthier lifestyles, and a greater ability to manage diabetes effectively. However, despite these benefits, these socioeconomic factors did not uniformly correlate with lower depressive symptoms. This observation highlights the complexity of the psychological impacts of diabetes, which appear to transcend economic and educational advantages [37, 40].

Furthermore, the study revealed that while insulin use was not statistically associated with depressive symptoms, it was linked to lower HRQoL scores. This finding suggests that additional research is needed to understand how diabetes treatment regimens, particularly insulin therapy, impact psychological well-being.

Overall, this study contributes valuable data to the existing knowledge base regarding the HRQoL among T2DM patients in a resource-constrained setting, emphasizing the intertwined roles of physical health, MH, and socioeconomic factors. The strong negative association between depressive symptoms and HRQoL highlights the necessity of incorporating MH support into diabetes care plans. Interventions should prioritize routine MH screenings and tailored psychological support to mitigate the impact of depressive symptoms on physical and emotional well-being. For older adults, interventions addressing age-related declines in physical health should integrate MH strategies to maximize overall quality of life.

The study's findings hold implications beyond the Hanoi population, as they contribute to the broader discourse on diabetes management in low- and middle-income countries. Future research should explore longitudinal data to assess causality and intervention effectiveness in improving both physical and MH outcomes among diabetic populations.

This study had several limitations. Firstly, this study's findings are based on data collected from a single medical center in Hanoi, which might not fully represent the diverse socioeconomic and healthcare environments experienced by T2DM patients across different regions of Vietnam, particularly those in rural areas. Secondly, the research's cross-sectional design further limits the ability to infer causality between T2DM, depressive symptoms, and HRQoL. To better understand the directionality and temporal changes of these relationships, future studies should consider longitudinal designs that can provide more definitive evidence of causation and potentially reveal different dynamics over time.

## 5. Conclusion

In conclusion, this study provides valuable insights into the impact of depressive symptoms on HRQoL among T2DM patients in Hanoi, Vietnam. The findings highlight that depressive symptoms significantly diminish HRQoL, particularly in PF and overall quality of life. These results underscore the importance of integrating MH assessments and interventions into routine diabetes care to enhance patient outcomes.

However, the conclusions must be interpreted within the context of the study's limitations. The cross-sectional design precludes causal inferences, and the single-center setting may limit the generalizability of the findings to broader populations, particularly those in rural or socioeconomically diverse areas of Vietnam. Future research should employ longitudinal designs and include multicenter studies to validate and expand upon these findings.

While this study advances understanding of the interplay between depressive symptoms and HRQoL in a resource-limited setting, further research is necessary to develop effective, scalable, and culturally sensitive interventions that address both the physical and psychological burdens of T2DM.

## Figures and Tables

**Table 1:** Level of depressive symptoms among diabetic patients.

	**Frequency (** **n** = 516**)**	**Percentage (%)**
Normal	283	54.8
Mild	186	36.0
Moderate	39	7.6
Moderate severe	7	1.4
Severe	1	0.2

	**Mean**	**SD**
PHQ-9 score	5.0	2.9

*Note:* Table 1 is reproduced from Tran Kien et al. [25].

**Table 2:** Characteristics among diabetic patients.

**Characteristic**	**Total (** **N** = 516**)**	**With depressive symptoms (** **N** = 231**)**	**Without depressive symptoms (** **N** = 285**)**	**p** ** value**
*Sociodemographic factors*				
Gender (male)				0.53
Male	220 (42.6%)	95 (41.1%)	125 (43.9%)	
Female	296 (57.4%)	136 (58.9%)	160 (56.1%)	
Age (mean, SD)	67 (9.0)	66 (8.0)	68 (9.0)	*0.03*⁣^∗^^b^
Education level				
Below secondary school	93 (18.0%)	44 (19.1%)	49 (17.2%)	0.42
Secondary school	251 (48.6%)	117 (50.7%)	134 (47.0%)	
Higher than secondary	172 (33.3%)	70 (30.3%)	102 (35.8%)	
Occupation				0.30
Employed	163 (31.6%)	70 (30.3%)	93 (32.6%)	
Retired	326 (63.2%)	145 (62.8%)	181 (63.5%)	
*Clinical characteristics*				
BMI (mean, SD)	22.8 (2.8)	22.8 (2.7)	22.9 (2.8)	0.70
Comorbidities	382 (90.3%)	208 (88.1%)	174 (93.1%)	0.09
Disease duration (mean years, SD)	8.0 (6.5)	8.6 (6.9)	7.6 (6.1)	0.13
Forgot medication last month	46 (9.5%)	22 (8.2%)	24 (11.2%)	0.28
Insulin injection	97 (18.8%)	48 (20.8%)	49 (17.2%)	0.34
*Behavioural factors*				
Smoking				0.27
Never	400 (77.5%)	186 (80.5%)	214 (75.1%)	
Former	64 (12.4%)	23 (10.0%)	41 (14.4%)	
Current	52 (10.1%)	22 (9.5%)	30 (10.5%)	
Drinking alcohol				*0.04*⁣^∗^^a^
Never	392 (76.9%)	186 (81.9%)	206 (72.8%)	
Former	34 (6.7%)	13 (5.7%)	21 (7.4%)	
Current	84 (16.5%)	28 (12.3%)	56 (19.8%)	

*Note:* Italicized values are significant at *p* < 0.05.

^a^From Pearson's chi-square test *p* value.

^b^From Mann–Whitney *U* test *p* value for nonparametric variables.

⁣^∗^*p* < 0.05.

**Table 3:** Quality of life component scores of study participants between T2DM patients with and without depression symptoms.

	**Total (** **n** = 516**)**		**With depressive symptoms (** **n** = 233**)**		**Without depressive symptoms (** **n** = 283**)**		**p** ** value**
	**Mean**	**SD**	**Mean**	**SD**	**Mean**	**SD**	
Physical functioning	48.58	20.90	45.48	20.29	51.21	20.47	*0.020*⁣^∗^
Role limitations due to physical health	33.64	35.27	29.51	35.07	37.11	35.18	*0.015*⁣^∗^
Role limitations due to emotional problems	52.19	44.63	53.93	45.43	51.01	44.04	0.460
Vitality	65.74	9.54	63.91	9.84	67.19	9.04	*<0.001*⁣^∗∗∗^
Mental health	69.94	13.23	67.64	13.59	71.75	12.64	*< 0.001*⁣^∗∗∗^
Social functioning	66.31	15.91	66.31	16.93	66.39	15.08	0.956
Bodily pain	73.79	25.10	69.94	24.64	76.78	25.08	*0.002*⁣^∗∗^
General health perceptions	83.95	39.78	85.78	38.28	82.82	40.80	0.399
Physical composite score	59.88	19.87	57.68	19.68	61.98	19.68	*0.014*⁣^∗^
Mental composite score	63.43	12.19	62.95	12.91	64.08	11.66	0.294
Overall quality of life	61.54	14.30	60.31	14.30	63.03	14.20	*0.031*⁣^∗^

*Note:* Italicized values are significant at *p* < 0.05, *p* < 0.01, and *p* < 0.001 from chi-square *t*-test results.

⁣^∗^*p* < 0.05.

⁣^∗∗^*p* < 0.01.

⁣^∗∗∗^*p* < 0.001.

**Table 4:** Correlation between health-related quality of life scores and other factors: correlation table (Pearson's rank correlation coefficient).

	**Physical composite score**	**Mental composite score**	**PHQ-9**	**BMI**	**Age**	**Duration**
Overall quality of life	*0.898*⁣^∗∗∗^	*0.806*⁣^∗∗∗^	*−0.133*⁣^∗∗^	−0.059	*−0.305*⁣^∗∗∗^	−0.049
Physical composite score	—	0.511⁣^∗∗∗^	*−0.145*⁣^∗∗^	−0.064	*−0.276*⁣^∗∗∗^	−0.037
Mental composite score		—	*−0.094*⁣^∗^	−0.016	*−0.253*⁣^∗∗∗^	−0.043
PHQ-9			—	0.006	*0.131*⁣^∗^	0.039
BMI				—	0.009	−0.029
Age					—	*0.207*⁣^∗∗∗^
Duration						—

*Note:* Italicized values are significant at *p* < 0.05, *p* < 0.01, and *p* < 0.001 from Spearman's test results.

⁣^∗^*p* < 0.05.

⁣^∗∗^*p* < 0.01.

⁣^∗∗∗^*p* < 0.001.

**Table 5:** Factors associated with HRQoL among diabetic patients.

	**Physical component score**		**Mental component score**		**Overall quality of life**	
	**Coef**	**95% CI**	**Coef**	**95% CI**	**Coef**	**95% CI**
Sex (male vs. female)	2.028	−4.011; 3.969	−1.063	−3.607; 1.481	−0.542	−3.438; 2.354
Age	−0.734⁣^∗∗∗^	−0.993; −0.475	−0.371⁣^∗∗∗^	−0.536; −0.206	−0.552⁣^∗∗∗^	−0.740; −0.364
Educational attainment (secondary or above vs. under secondary)	5.951⁣^∗^	0.631; 11.270	1.980	−1.412; 5.371	3.965⁣^∗^	0.104; 7.826
Occupation (employed vs. retired and unemployed)	6.624⁣^∗∗^	2.091; 11.157	2.932⁣^∗^	0.041; 5.822	4.778⁣^∗∗^	1.488; 8.068
BMI	0.008	−0.695; 0.711	−0.068	−0.516; 0.380	−0.030	−0.540; 0.480
Diabetes duration	0.006	−0.021; 0.034	−0.007	−0.024; 0.010	0.000	−0.020; 0.029
PHQ-9	−1.078⁣^∗∗^	−1.762; −0.395	−0.291	−0.727; 0.145	−0.685⁣^∗∗^	−1.181; −0.188
Medical adherence (no vs. yes)	−2.665	−9.455; 4.125	−4.696⁣^∗^	−9.025; −0.367	−3.681	−8.609; 1.248
Comorbidity (yes vs. no)	5.176	−1.331; 11.684	1.009	−3.140; 5.158	3.093	−1.631; 7.816
Insulin injection (yes vs. no)	4.303	−0.535; 9.142	1.051	−2.034; 4.136	2.677	−0.835; 6.189

⁣^∗^*p* < 0.05.

⁣^∗∗^*p* < 0.01.

⁣^∗∗∗^*p* < 0.001.

## Data Availability

The data supporting this study's findings are available from the corresponding author upon reasonable request. Appropriate measures have been taken to ensure participant confidentiality, and any shared data will be deidentified. Researchers seeking further details are welcome to contact the corresponding author at the address listed in the manuscript.
